# Emergency thoracoscopic management of early ablation-tract bronchopleural fistula after low-power lung microwave ablation: a case report

**DOI:** 10.1186/s13019-026-04526-7

**Published:** 2026-07-20

**Authors:** Weike Lv, Jiewen Deng, Dongjie Feng

**Affiliations:** Department of Thoracic Surgery, The Affiliated Cancer Hospital of Nanjing Medical University, Jiangsu Cancer Hospital, Nanjing, Jiangsu P.R. China

**Keywords:** Microwave ablation, Bronchopleural fistula, Pneumothorax, Empyema, Video-assisted thoracoscopic surgery

## Abstract

**Background:**

Microwave ablation (MWA) is an established local therapy for selected pulmonary nodules in patients who are poor candidates for surgery. Bronchopleural fistula (BPF) is a rare but serious complication after lung ablation.

**Case presentation:**

A 74-year-old woman with prior bilateral thoracic interventions and impaired pulmonary reserve underwent CT-guided percutaneous MWA (30 W for 4 min) for a right upper lobe ground-glass nodule on post-ablation day (PAD) 0. On PAD2, chest radiography showed a large right pneumothorax with more than 50% right lung compression, and right chest tube drainage was promptly performed with symptomatic improvement. On PAD5, she developed worsening chest tightness and rapidly increasing fever; in the context of recurrent pneumothorax and poor lung re-expansion despite chest drainage, urgent CT showed findings highly suggestive of BPF with pleural infection/empyema, including an air-fluid cavity along the ablation tract. Emergency VATS was performed on the same day. Intraoperatively, a thick-walled purulent necrotic cavity was identified along the ablation tract and communicated with a distal branch of the anterior segmental bronchus. Thoracoscopic adhesiolysis, decortication, debridement, partial pulmonary resection of the fistula-bearing lung tissue, pleural irrigation, and chest tube drainage were performed. The pneumothorax resolved, lung re-expansion was satisfactory, and the patient recovered and was discharged. At 3 months, no clinical evidence of recurrent fistula or need for reintervention was observed, and 1-month CT showed no definite radiologic evidence of recurrent BPF, empyema, or local progression.

**Conclusions:**

This case highlights that BPF may occur early even after low-power lung MWA. In a post-MWA pneumothorax patient, new fever, poor lung re-expansion despite chest drainage, and a CT air-fluid cavity along the ablation tract should raise suspicion for MWA-tract-related BPF. In selected severe cases with pleural infection/empyema and a non-expanding lung, prompt thoracoscopic source control may be considered, but a single case cannot define a universal management algorithm.

## Background

Percutaneous microwave ablation (MWA) has emerged as a minimally invasive option for selected lung tumors, especially in patients with compromised pulmonary reserve or those who are poor candidates for resection [1]. While pneumothorax and hemorrhage are more common, bronchopleural fistula (BPF) is a rare complication that can rapidly progress to pleural infection, empyema, and lung collapse [2, 3]. We present a case of early ablation-tract-related BPF following low-power MWA that required emergency video-assisted thoracoscopic surgery (VATS) for surgical source control.

## Case presentation

**Fig. 1:**
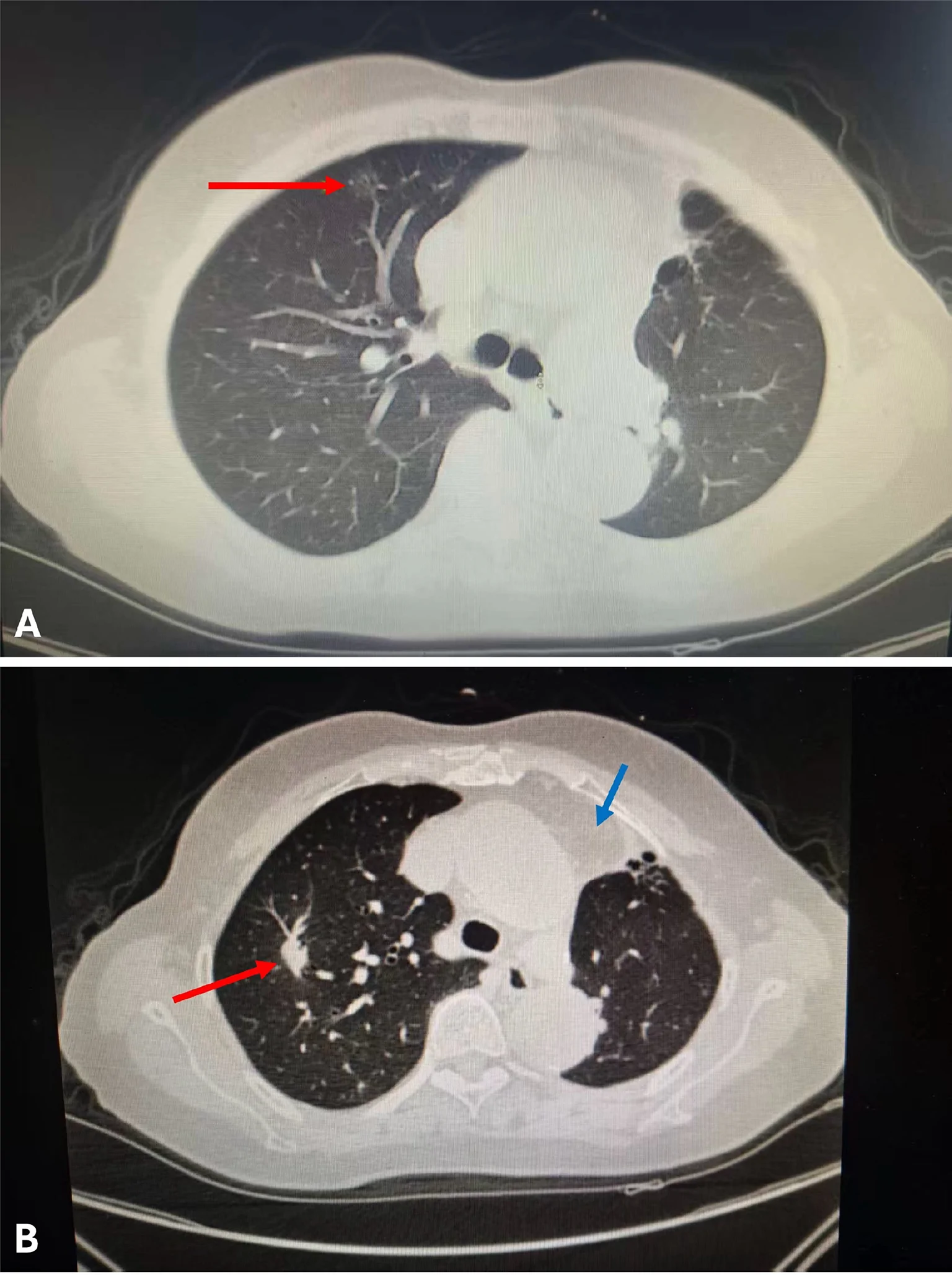
Pre-procedural chest CT (lung window). (**A**) Target ground-glass nodule in the anterior segment of the right upper lobe (red arrow). (**B**) Prior right lung cryoablation scar (red arrow) and postoperative absence of the left upper lobe after lobectomy (blue arrow)

A 74-year-old woman was admitted for management of a 1.2 cm ground-glass nodule in the anterior segment of the right upper lobe, detected on routine follow-up CT (Fig. 1A). Her history included left upper lobectomy via VATS for adenocarcinoma 5 years earlier and subsequent cryoablation of a contralateral pulmonary nodule (Fig. 1B). She was an ex-smoker with a 20-pack-year history. Pulmonary function testing showed moderate mixed ventilatory impairment, with an FEV1 of 1.35 L (62% predicted), FVC of 2.10 L (75% predicted), FEV1/FVC ratio of 64%, and DLCO of 58% predicted. Given the small lesion size, prior bilateral thoracic interventions, limited pulmonary reserve, and the patient’s preference to avoid another formal resection, percutaneous ablation was considered a more appropriate local treatment than repeat surgery, while stereotactic radiotherapy was not prioritized in the multidisciplinary discussion. Pre-ablation biopsy was not performed because the lesion was a small ground-glass nodule with only a limited solid component, and management was based on radiologic suspicion in the context of her prior lung cancer history and multidisciplinary consensus.

On December 4, 2025, defined as PAD0, MWA was performed under local anesthesia using a Surgnova Dophi 150E ablation system. Under CT guidance, ablation was delivered at 30 W for 4 min, creating an approximately 2 × 3 cm ablation zone. Immediate post-procedural CT showed no pneumothorax, hemothorax, or other acute complication, and the patient returned to the ward in stable condition.

**Fig. 2:**
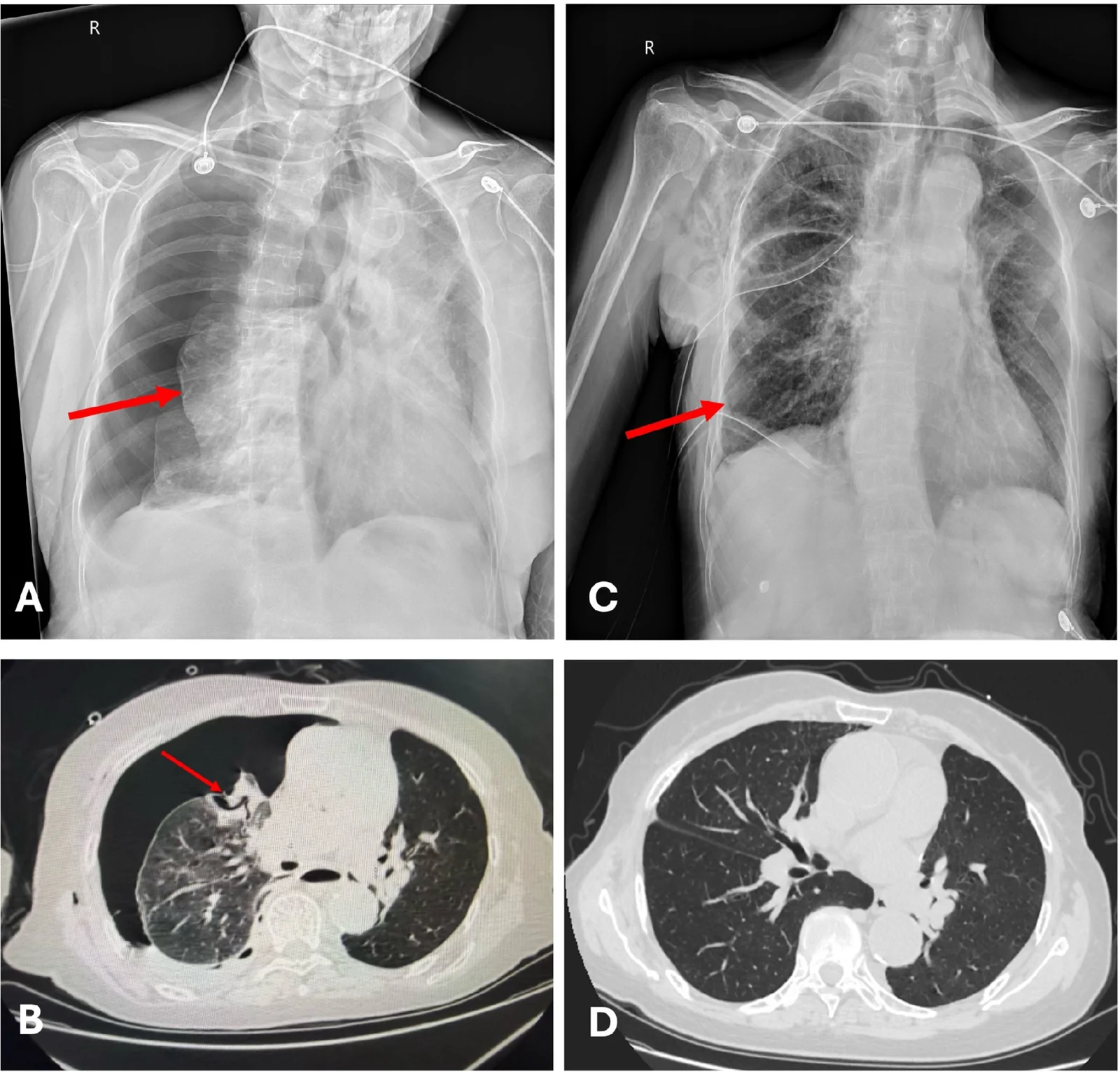
Radiographic progression and short-term follow-up. (**A**) Chest radiograph on PAD2 showing a large right pneumothorax with more than 50% right lung compression (arrow). (**B**) Chest CT on PAD5 showing an air-fluid cavity with surrounding increased opacity along the MWA tract, highly suggestive of BPF with pleural infection/empyema (arrow). (**C**) Postoperative chest radiograph after VATS showing satisfactory right lung re-expansion (arrow). (**D**) One-month follow-up chest CT showing expected postoperative changes with good right lung re-expansion and no definite radiologic evidence of recurrent BPF

On PAD2 (December 6, 2025), chest radiography showed a marked right pneumothorax with more than 50% compression of the right lung (Fig. 2A). Right chest tube drainage was promptly performed, and the patient’s symptoms improved. On PAD3 (December 7, 2025), repeat chest radiography showed approximately 35% residual compression of the right lung and patchy right lung opacity, interpreted as post-treatment change; chest drainage was therefore continued.

On PAD5 (December 9, 2025), the patient reported worsening chest tightness and rapidly developed fever. Urgent chest radiography showed recurrent right lung compression of approximately 45%. Bronchoscopy was performed before the emergency CT mainly for airway toilet. Thin light-yellow secretions were aspirated, but no definite fistula opening was visualized, likely because the communication was distal to the anterior segmental bronchial orifice and was later confirmed intraoperatively to involve a distal branch of the anterior segmental bronchus. No bronchoscopic images were available because a simple bronchoscope without video-recording capability was used.

Emergency chest CT obtained on PAD5 showed findings highly suggestive of BPF with pleural infection/empyema, including an air-fluid cavity along the MWA tract with surrounding inflammatory change, pleural air-fluid collection, poor lung expansion, and associated atelectatic change (Fig. 2B). Given the rapid clinical deterioration, poor lung re-expansion despite chest drainage, CT evidence of an ablation-tract air-fluid cavity suspicious for BPF, and the need for urgent surgical management, emergency VATS was performed on the same day as the CT diagnosis. Because of the urgent need for surgery, pleural fluid and blood cultures were not obtained preoperatively. Purulent material and necrotic tissue were encountered intraoperatively but were not sent for microbiological culture.

**Fig. 3:**
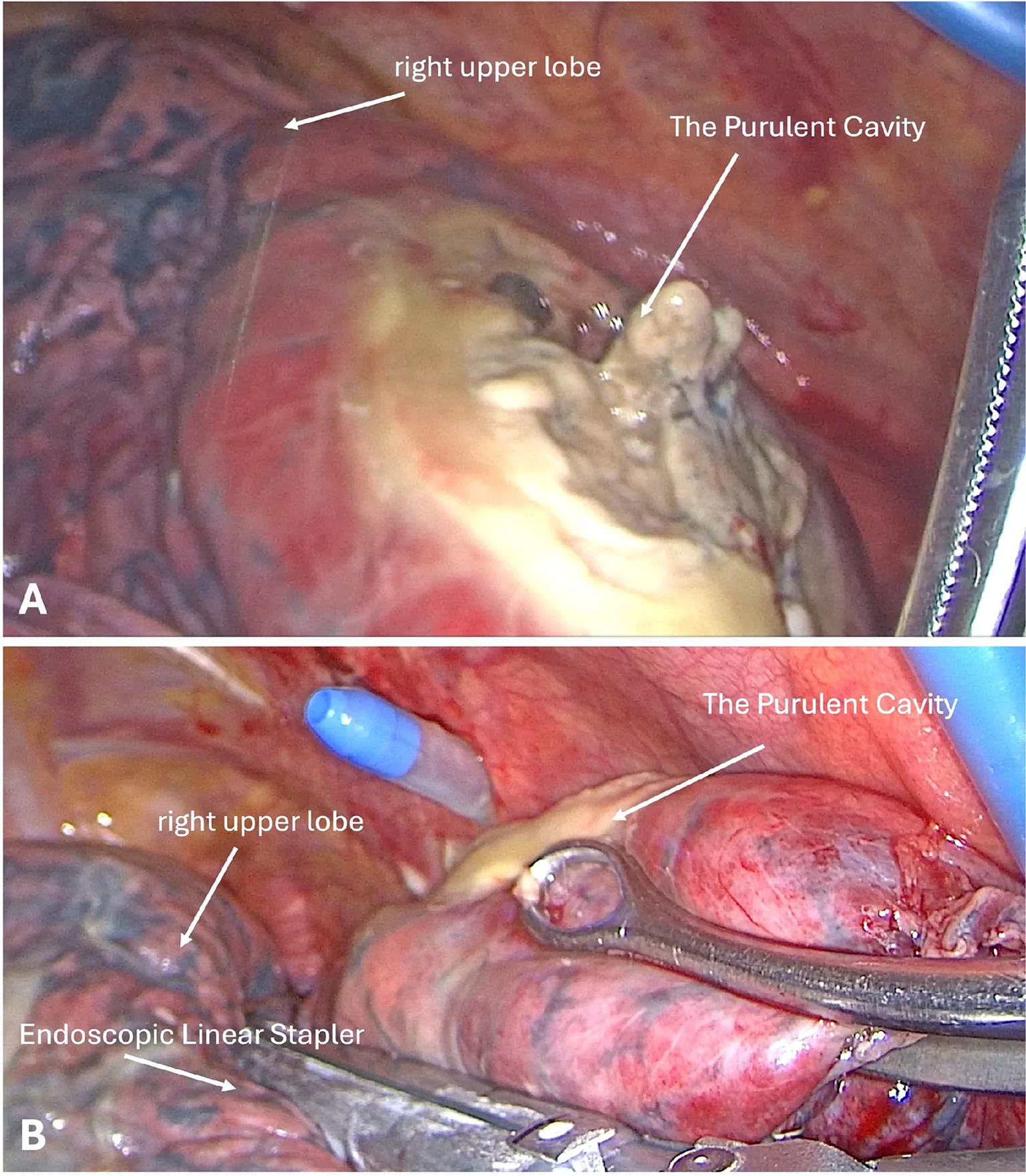
Intraoperative VATS findings. (**A**) Thick-walled purulent necrotic cavity along the ablation tract in the anterior segmental region of the right upper lobe (arrow). (**B**) Resection of the cavity and fistula-bearing lung tissue with an endoscopic linear stapler (arrow)

**Fig. 4:**
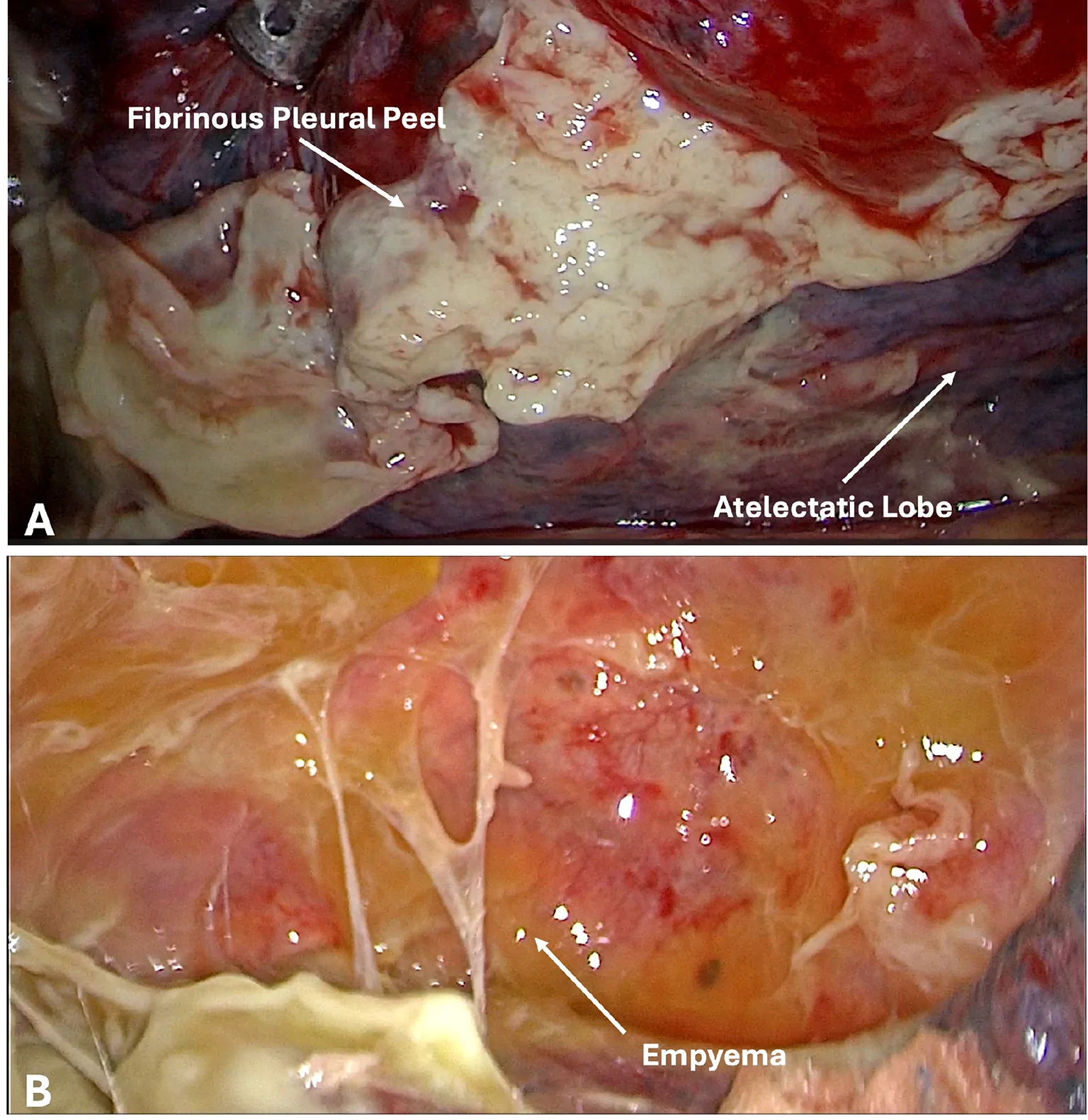
Intraoperative findings of empyema and pleural peel. (**A**) Fibrinous pleural peel encasing the atelectatic lobe (arrow). (**B**) Frank purulent material within the pleural space, confirming empyema (arrow)

Emergency VATS was performed under general anesthesia. Thoracoscopic exploration revealed a thick-walled purulent necrotic cavity along the ablation tract in the anterior segmental region of the right upper lobe (Fig. 3A). Intraoperative exploration further confirmed communication between this cavity and a distal branch of the anterior segmental bronchus. The middle and lower lobes were markedly collapsed and encased by a fibrinous pleural peel, and frank empyema was present (Fig. 4A and B). Thoracoscopic adhesiolysis, decortication, and debridement were performed to facilitate lung re-expansion and clear the infected pleural space. The necrotic cavity and fistula-bearing lung tissue in the anterior segmental region were removed by thoracoscopic partial pulmonary resection using an endoscopic stapler (Fig. 3B). The pleural cavity was soaked with diluted povidone-iodine solution and then thoroughly irrigated. Two chest tubes were placed for postoperative drainage.

The postoperative course was uneventful, with resolution of the pneumothorax and satisfactory lung re-expansion on imaging (Fig. 2C). Routine pathology of the resected specimen showed extensive degenerative necrotic tissue with scant residual epithelioid cellular remnants, consistent with post-treatment regression change in the clinical context. The patient was discharged 7 days after VATS.

Follow-up was performed at 1 month and 3 months after surgery. The patient reported near-normal daily activity without oxygen requirement and only mild intermittent incisional pain. She denied recurrent dyspnea, chest tightness, or the need for repeat drainage or further thoracic procedures. A chest CT obtained 1 month after surgery showed expected postoperative changes with good right lung re-expansion, a small right pleural effusion, near-complete absorption of the previous ectopic gas, and no definite radiologic evidence of recurrent BPF, empyema, or local progression in the treated right lung (Fig. 2D). The patient recalled one self-limited febrile episode approximately 2 months postoperatively that resolved after self-medication and was not medically evaluated; therefore, its cause remained uncertain. No rehospitalization or further intervention was required during the available follow-up.

## Discussion and conclusions

MWA is a useful alternative to resection in selected high-risk patients, particularly those with limited pulmonary reserve or prior thoracic interventions [1]. In the present case, MWA was selected after multidisciplinary discussion because of the small ground-glass lesion, the patient’s prior bilateral thoracic procedures, impaired pulmonary reserve, and preference for a less invasive local treatment. Although common complications of lung MWA include pneumothorax and pleural effusion, BPF is rare but potentially life-threatening [4]. This case is notable because clinically significant BPF developed early after low-power ablation parameters (30 W for 4 min), emphasizing that severe airway-pleural communication can occur even when relatively conservative energy settings are used [5].

The central case-specific insight is the probable ablation-tract mechanism. The intraoperative finding of a thick-walled purulent necrotic cavity along the MWA tract that communicated with a distal branch of the right upper lobe anterior segmental bronchus supports a plausible sequence of tract-related airway injury, thermal necrosis, distal bronchial communication, and secondary pleural contamination. Prior thoracic interventions may have increased procedural complexity and may have contributed to pleural adhesions and impaired regional reserve, although causality cannot be established from a single case [6, 7].

The diagnostic message is also case-specific. In post-MWA patients, pneumothorax alone is common and does not necessarily indicate BPF. However, in this patient, the combination of recurrent or persistent lung collapse despite chest drainage, new fever, and a CT air-fluid cavity along the ablation tract raised concern for MWA-tract-related BPF with pleural infection. This pattern may help distinguish uncomplicated post-ablation pneumothorax from a more severe ablation-tract fistula complicated by pleural infection/empyema.

The timing of surgery in this case was not based on a fixed duration of failed conservative therapy. Chest drainage was initially performed for the PAD2 pneumothorax. Emergency VATS was undertaken on PAD5 after clinical deterioration and CT findings strongly suggested BPF with pleural infection and a non-expanding lung. Thus, the operative decision was driven by the need for diagnostic confirmation and prompt source control rather than by an arbitrary observation period.

VATS served both diagnostic and therapeutic roles in this patient. It confirmed the ablation-tract cavity and distal bronchial communication, allowed adhesiolysis and decortication of fibrinous pleural peel, enabled debridement and limited removal of fistula-bearing necrotic lung tissue, and provided pleural irrigation and drainage. In this case, prompt thoracoscopic source control was followed by resolution of the pneumothorax, satisfactory lung re-expansion, and short-term clinical recovery. These observations should be interpreted as case-specific and hypothesis-generating rather than as proof of a general treatment pathway [8–10].

This case highlights that early BPF may occur after low-power lung MWA and that fever, poor lung re-expansion despite chest drainage, and a CT air-fluid cavity along the ablation tract should prompt consideration of MWA-tract-related BPF. For selected severe cases with pleural infection/empyema and a non-expanding lung, prompt thoracoscopic exploration and source control may be considered as part of individualized management.

This report has several limitations. First, pre-ablation biopsy was not performed, and the salvage specimen showed predominantly post-ablation necrosis; therefore, the treated nodule lacked definitive pre-ablation histologic confirmation. Second, pleural fluid and blood cultures were not obtained before emergency surgery, and intraoperative purulent material and necrotic tissue were not sent for microbiological culture; therefore, microbiological confirmation and pathogen identification were unavailable. Third, bronchoscopy was performed with a simple non-recording bronchoscope, so bronchoscopic image documentation was unavailable, and the fistula opening was not visualized endoscopically. Fourth, follow-up was short, with imaging available at 1 month and clinical follow-up at 3 months. Finally, the single-case nature of this report limits generalizability and cannot define a universal threshold or algorithm for surgical intervention after MWA-related BPF.

## Data Availability

The data supporting the findings of this case report are included within this article.

## Abbreviations

BPF: Bronchopleural fistula
MWA: Microwave ablation
VATS: Video-assisted thoracoscopic surgery
FEV1: Forced expiratory volume in 1 s
FVC: Forced vital capacity
DLCO: Diffusing capacity for carbon monoxide
PAD: Post-ablation day
